# A Comparison of the Gut Microbiome of Two Sympatric Macropods Along an Urbanisation Gradient in Tasmania

**DOI:** 10.1111/1758-2229.70359

**Published:** 2026-05-12

**Authors:** Hanh K. D. Nguyen, Penelope J. Jones, Dave Kendal, Shane M. Powell, Kelsie Raspin, Jo Dickinson, Emily J. Flies

**Affiliations:** ^1^ School of Geography, Planning and Spatial Sciences, Geography Building University of Tasmania Sandy Bay TAS Australia; ^2^ Healthy Landscapes Research Group University of Tasmania Sandy Bay TAS Australia; ^3^ Menzies Institute for Medical Research University of Tasmania Hobart TAS Australia; ^4^ Future in Nature Pty Ltd VIC Australia; ^5^ Tasmanian Institute of Agriculture (TIA), Life Sciences Building University of Tasmania Sandy Bay TAS Australia

## Abstract

This study investigates the gut flora of the red‐necked wallaby (*Notamacropus rufogriseus rufogriseus*) and the Tasmanian pademelon ( *Thylogale billardierii* ) in the small city of Hobart, Tasmania, Australia. Faecal samples were collected from greenspaces across the Greater Hobart region. These greenspaces were chosen in areas with different human population densities. DNA was extracted from these samples, and targeted sequencing of the bacterial 16S ribosomal RNA gene was performed to understand the bacterial community. Our results showed that despite the many shared ecological traits between the two species, their gut microbiome displayed different responses to urban living. Alpha and beta diversity were significantly different across the urban gradient for Bennett's wallaby, but not for Tasmanian pademelon. Relative composition for both species was different across the urbanisation gradient. Some bacterial taxa associated with nutrient processing showed the clearest changes. The conclusion of this research is that living in cities can affect the gut microbiome of these two marsupial herbivores. More studies are needed to determine whether this has implications for the health of these species.

## Graphical abstract

Our study is the first to explore how macropod gut flora vary across levels of urbanisation, exploring the faecal microbiomes of the red‐bellied pademelon ( *Thylogale billardierii* ) and the red‐necked wallaby (*Notamacropus rufogriseus rufogriseus*). There is some evidence of urbanisation's impacts on the macropod microbiome, although there appear to be nuances across species and some non‐linear relationships along a human population density gradient in a small city.

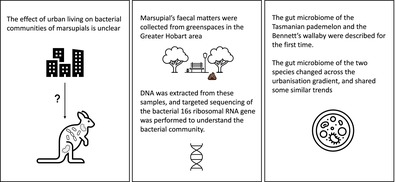

## 1 Introduction

Advances in high throughput sequencing have created an opportunity for research into the microbial communities of specific niches in many organisms, amongst which the gut flora is particularly important. In both humans and animals, gut flora has been found to contribute to their host's homeostasis and take part in regulating many internal systems and functions (Willem et al. 2022; Upadhaya and Kim 2022; Cho et al. 2022). Many diseases are linked to gut microbiota dysbiosis (Hou et al. 2022; Degruttola et al. 2016; Madhogaria et al. 2022). In animals, the gut microbiota can be influenced by external factors, especially diets (Beam et al. 2021; Ringø et al. 2016), and habitats (Chen et al. 2022; Drobniak et al. 2022; Grieneisen et al. 2019).

Urban areas are a rapidly expanding habitat type and thus are becoming an important and novel home to many wildlife species (Mckinney 2006; Boakes et al. 2024). Cities expose wildlife to challenges never before experienced in their evolutionary history, such as altered resources, new biotic and abiotic habitat conditions, high levels of pollution in water and air, and high levels of human disturbance (Murray et al. 2019; Markovchick‐Nicholls et al. 2008), which can all potentially lead to major shifts in their gut microbiome. Thus, understanding the various ways wildlife adapts and responds to city life, including through changes in their gut bacterial assemblage, will be key to effective conservation of urban‐living species.

However, the impact of urbanisation on microbial biodiversity is a complicated, on‐going field of research. A major challenge is that cities are complex mosaics with widely varying environmental variables (Hahs and Mcdonnell 2006). Reducing this mosaic to understand how and why urban living and urbanisation are impacting wildlife microbiomes is challenging. The impact of urbanisation on biodiversity is a complicated, on‐going field of research. Urban growth, urban population, and urban land uses have all been assessed as individual factors influencing the assemblages of plants, vertebrate animals, and invertebrate animals (Mckinney 2006; Łopucki et al. 2020; Bray and Wickings 2019; Teyssier et al. 2020; Phillips et al. 2018). However, cities are combinations of these factors, complicating what conclusion can currently be drawn.

Previous research on wildlife taxa including mammals, birds and insects has had mixed results—with some studies finding lower diversity in the microbiomes of urban individuals (Teyssier et al. 2020; Maraci et al. 2022), some finding higher diversity (Sugden et al. 2020; Berlow et al. 2021), and some finding no differences (Murray et al. 2020; Stephens et al. 2021). Some studies have reported a significant effect of urbanisation only on some genera and/or families of bacteria, rather than the overall studied microbiome (Stephens et al. 2021; Stothart and Newman 2021). Comparable studies on domestic animals have likewise found conflicting results in terms of the impact of urbanisation on host taxon microbiome diversity. For example, one study on the faecal microbiomes of domestic dogs growing up in big cities found that they were more diverse than those of dogs growing up in small cities and the countryside (Vilson et al. 2018). Another study found that skin microbiomes of domestic dogs were overall more homogenous in cities than in rural areas; further, the dogs who lived in cities were also more susceptible to allergic diseases (Lehtimäki et al. 2018). Apart from urbanisation, other factors such as microhabitats, stress, diets and fur colour have also been linked to the diversity and/or composition of urban wildlife microbiota (Nguyen et al. 2024).

In addition to the web of contrasting impacts that have been reported, another factor complicating our understanding of the impacts of urbanisation on animal microbiomes is the lack of comparability across many studies. This is due to differences in methodology such as various definitions of urbanisation, different targeted regions of the 16S rRNA gene, and different operational taxonomic unit grouping (Nguyen et al. 2024; Flies et al. 2020). To further complicate these explorations, cities are complex mosaics with widely varying environmental variables (Hahs and Mcdonnell 2006). Different bacterial taxa also present various reactions to the pressure of city living (Nguyen et al. 2024; Grierson et al. 2023). In this context, there is a clear need for further research to resolve when, and in what types of contexts, urbanisation affects wildlife microbiota.

Macropods are often overlooked in urban studies despite being abundant in many cities and towns in Australia (Fardell and Dickman 2023). Very few studies to date have investigated the microbiota of macropod species (O'Dea et al. 2021; Gulino et al. 2013) and none have investigated how their microbiomes might be influenced by urban living. The Greater Hobart area provides easy access to the habitats of these targeted species. Additionally, smaller cities (with a population less than ~300 k) are underrepresented in urban biodiversity research while large, well‐developed cities (> 1.76 M) are over‐represented (Kendal et al. 2020). Thus, studying an urban gradient in a less densely populated urban area—i.e. a ‘smaller city’ such as Hobart—helps elucidate the impact of this underexplored but commonly experienced—by wildlife and people—form of urbanisation, especially as small cities are more abundant and a more common way of urban living in every country (Grossmann and Mallach 2021; Frick and Rodríguez‐Pose 2018).

This study seeks to address these identified knowledge gaps. The specific aim of this research is to understand the impact of urban living on the composition and diversity of gut microbiomes of the Tasmanian pademelon ( *Thylogale billardierii* ) and the Bennett's wallaby (*Notamacropus rufogriseus rufogriseus*) in the small city of Hobart, Tasmania, Australia. Like other members of the family Macropodidae, these species are both foregut fermenters (Hume 1999; Smith 2009), they often occupy the same habitats and their diets overlap (Le Mar and Mcarthur 2005), thus allowing us to compare the impact of urbanisation on their gut microbiomes while controlling for some of the variability urban environments provide for different species. The ubiquity of these species across a population density gradient in the region (Garvey 2011; Driessen and Hocking 1992) makes them ideal species with which to explore the impact of urbanisation on wildlife gut microbiomes. Ours is the first study to examine the gut microbiome of these two Tasmanian macropod species in a non‐captive environment.

## 2 Methods

### 2.1 Study Area and Species

Tasmania is an island state of high endemism located to the south of mainland Australia. The state has a cool temperate oceanic climate with mild but distinctive summers and cool to cold winters (Chen et al. 2021). The Greater Hobart region comprises the city of Hobart—the capital city of Tasmania—and surrounding areas. Often characterised as a small city (Goodfellow and Prahalad 2022; Booth et al. 2021), The Greater Hobart region had a human population of 247,086 at the 2021 census and has an average population density of 150 people/km^2^ (ABS 2021).

The Tasmanian subspecies of the red‐necked wallaby, often referred to as Bennett's wallaby (*Notamacropus rufogriseus rufogriseus*), is a medium‐sized macropod that is abundant and widespread across the state (Garvey 2011). The red‐bellied pademelon, or Tasmanian pademelon ( *Thylogale billardierii* ), is a small‐sized macropod now found only in Tasmania and its islands (Rose et al. 2005). The species is highly abundant in the state, including in urban areas, with a stable population (Driessen and Hocking 1992). While both species are terrestrial herbivores, Bennett's wallaby is a grazer whose diet contains more than 70% grasses, and the Tasmanian pademelon is a browser whose diet contains more than 70% dicots (Grigg et al. 1989, Le Mar and McArthur, 2005, Arman and Prideaux 2015). In order to investigate the impact of urbanisation on these species, we collected fresh scat samples of free‐ranging pademelons and wallabies from multiple greenspaces within and around the Greater Hobart region, and examined the faecal microbiota using an Illumina metabarcoding approach targeting the bacterial 16S rRNA genes.

### 2.2 Field Collection

Faecal samples from both study species were collected from green spaces across the Greater Hobart area. Despite the faecal microbiome being only a proxy to the gut microbiome, it is non‐invasive and a good representative of the gut microbiome (Rajan et al. 2019; Yan, Sun, et al. 2019), making it suitable for this study.

**FIGURE 1:**
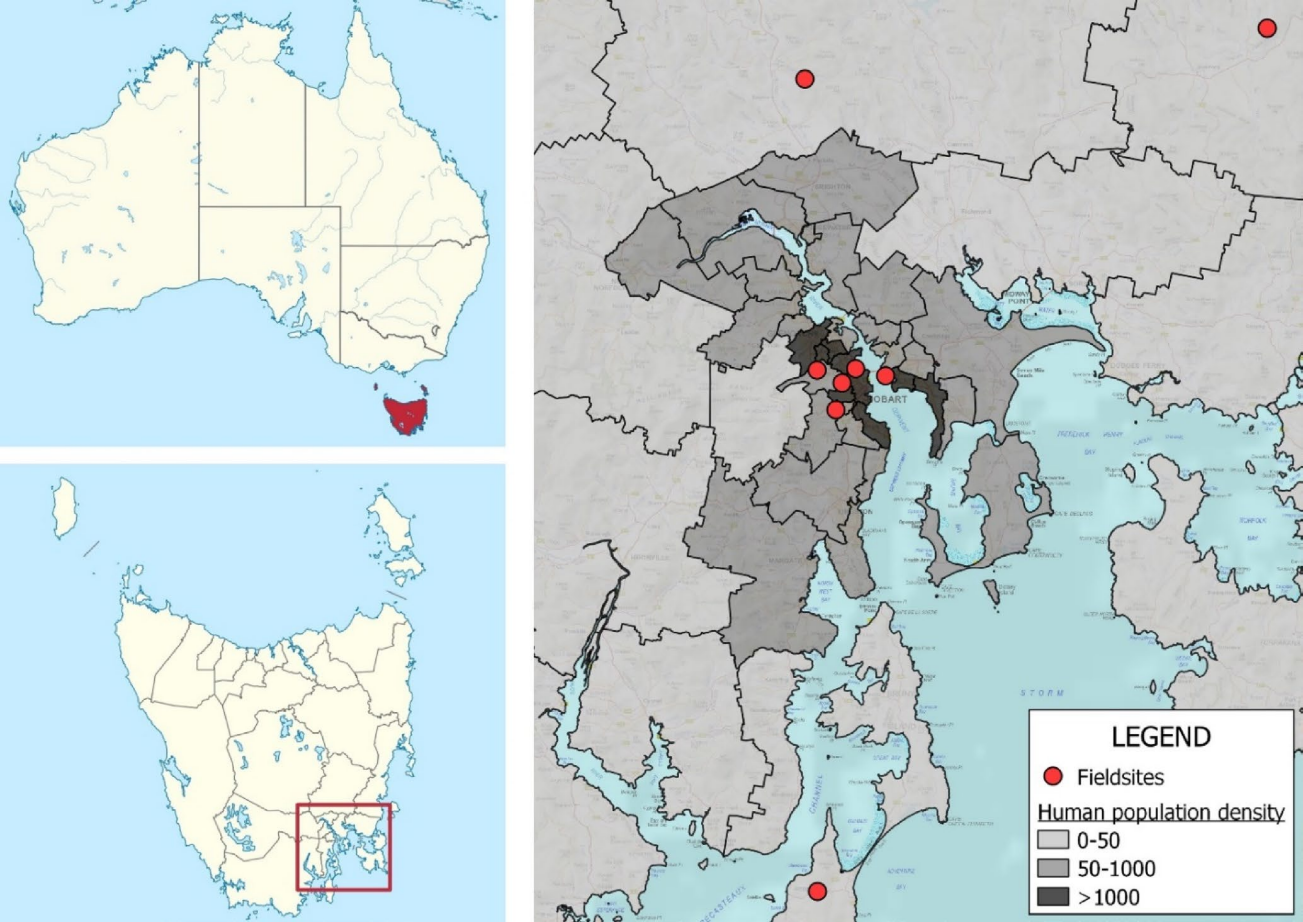
Geographical context of the sampling sites in the Greater Hobart area, Tasmania, Australia. Some features of the maps were adapted from online materials (NordNordWest 2009; TUBS 2011).

Using the Statistical Area Level 2 (SA2) data defined by the Australian Bureau of Statistics, eight parks and reserves were selected to provide representation along an urbanisation gradient defined by human population density. Specifically, we selected three sites in highly urbanised areas (D1 density > 1000 people/km^2^), two sites at a medium level of urbanisation (D2 density: 100–1000 people/km^2^), and three sites in non‐urban areas (D3 density < 50 people/km^2^) (Table 1, Figure 1).

**TABLE 1:** The Tasmanian greenspace sites surveyed for animal faecal samples, showing their human population density and number of samples.

Site	Urbanisation level	SA2 human population density (in people/km^2^)	Number of wallaby samples (*n* = 40)	Number of pademelon samples (*n* = 24)
Prosser River	D3	1.7	7	1
Chauncey Vale		2.3	5	2
Lutregala		6.6	4	5
Wellesley Park	D2	259	1	2
Rangeview Crescent		848	7	8
The Domain	D1	1180	5	0
Rosny Hill		1258	5	0
Leonard Wall		1621	6	6

The urban sites are managed by the Hobart and Clarence councils, while the non‐urban sites are managed by the Tasmanian Land Conservancy. All sites had extensive areas of grassland or managed lawn, suitable for pademelon and wallaby foraging, from which the samples were taken.

Fresh faecal samples were collected from each site for two consecutive days using metal chopsticks sterilised with 10% bleach and sterile gloves to avoid contamination with human DNA. Pademelon and wallaby faecal samples were collected, identified using the PooFlip which is a visual guide to the scats of Tasmanian Native Animals with life‐sized colour photographs and written descriptions (Wiltshire 2018). The differences in the size, morphology and consistency of these scats make them relatively easy to differentiate to the practiced eye, especially with the assistance of high‐quality guides such as the PooFlip. Each scat was stored in a 50 mL falcon tube with 100% ethanol and frozen at −20°C until extraction (Hale et al. 2015).

### 2.3 Microbiome Analysis

To characterise the microbial communities, we sequenced the 16S rRNA gene of bacteria by targeting the V3‐V4 region. The 16S rRNA gene is a common molecular marker in bacterial phylogeny and taxonomy studies. It is present in all bacteria, is very similar within the same genus and species with highly conserved function and constitutes the most informative portions for taxonomic classification for bacteria (Srinivasan et al. 2015; Janda and Abbott 2007). Sequencing 16S rRNA gene allows for accurate phylogeny placement through precise identification. We used primer pair 341F/805R (Klindworth et al. 2013) which were found to yield highly reproducible results (Thijs et al. 2017).

#### 2.3.1 DNA Extraction

The following protocol, similar to Tegart et al. (2024), was adapted for this study.

DNA was extracted in duplicate from each of the samples. The middles of the scat pellets were taken to minimise the influence of the ground microbiome. Samples were weighed and then put into 2‐mL tubes with 1 mL cetyltrimethylammonium (CTAB) buffer (1 M Tris‐Cl pH 8.0, 0.5 M ethylenediaminetetraacetic acid (EDTA) pH 8.0, 5 M Sodium Chloride, 2% (w/v) hexadecyltrimethylammonium bromide, 2% (w/v) polyvinylpolypyrrolidone 40) and one tungsten carbide bead. The samples were pulverised using a Qiagen TissueLyser II at 30 Hz for 5 min, checking for breakage at half time.

The mixture was incubated at 65°C with light shaking for 30 min. Five hundred millilitres of phenol:chloroform:isoamyl alcohol (25:24:1, v/v) was added. The mixture was thoroughly vortexed and then centrifuged at 14000 rpm for 5 min at 4°C. The top layer was then transferred to a new tube, to which another 500 mL of phenol:chloroform:isoamyl alcohol (25:24:1, v/v) was added. After vortexing, this mix was centrifuged at maximum speed for 10 min at 4°C. Then, the top layer was moved to a new tube. After 60 μL of 3 M sodium acetate was added, the mixture was incubated at room temperature for 5 min, then 700 μL of ice‐cold 100% ethanol alcohol was added.

The sample was left in freezer overnight to precipitate. Upon removal, tubes were centrifuged at maximum speed for 15 min at 4°C then supernatant was discarded. The DNA was washed by adding 500 μL of 75% ethanol, vortexed then centrifuged at maximum speed for 10 min at 4°C. This step was repeated twice with supernatant discarded each time. Tubes were left in a BioSafety Hood to dry. The extraction was purified using AMPure XP Bead‐Based Reagent by Beckman Coulter. DNA was resuspended in 30 μL of autoclaved deionised (milliQ) water in a heat bath for an hour then frozen until used.

#### 2.3.2 16S rRNA Gene Amplicon Sequencing

Aliquots from the DNA extracts were used for amplification of the V3‐V4 region of the microbial 16S rRNA gene using the primer pair Bakt_341F (CCTACGGGNGGCWGCAG) and Bakt_805R (GACTACHVGGGTATCTAATCC) (Klindworth et al. 2013; Thijs et al. 2017).

For the first round, the reaction mix contained: 1.2 μL DNase‐free water, 0.2 μL bovine serum albumin, 5.0 μL AmpliTaq Gold 360 Master Mix (Thermo Fisher), 0.8 μL of each Amplicon PCR Reverse Primer (10 μM) and Amplicon PCR Forward Primer (10 μM), and 2 μL of DNA template. PCR was performed in a thermal cycler using the following program: at 95°C for 5 min, then cycling was carried out as follows: 95°C for 30 s, 62°C for 30 s, and 72°C for 30 s for 35 cycles. A final elongation was performed at 72°C for 7 min.

For the second round, the template resulting from the first round was spiked with P5 and P7 Illumina sequencing adapters as follows: 1.0 μL of PCR grade water, 5.0 μL AmpliTaq Gold 360 Master Mix, 1 μL of i5 primer and 1 μL of i7 primer, and 2 μL of DNA template diluted to 0.5 ng/μL. Denaturation of DNA was performed at 95°C for 10 min, then cycling was carried out as follows: 95°C for 30 s, 55°C for 30 s, and 72°C for 30 s for 10 cycles. A final elongation was performed at 72°C for 7 min and held at 4°C.

The PCR product was pooled and purified using AMPure XP Bead‐Based Reagent. Quality checking was done using Tapestation sizing. Paired‐end sequencing reads were generated on the MiSeq platform (Illumina, San Diego, CA, USA) using MiSeq Reagent Kit v3 (2 × 300 bp paired‐end). Demultiplexing was done using Illumina software.

### 2.4 Statistical Analysis

Statistical analyses were performed in R 4.2.3 (R Core Team 2021).

Bioinformatic analysis was carried out following the Australian Microbiome standard amplicon workflow (Bissett et al. 2016). The resulting sequences were then denoised to zero radius Operational Taxonomic Units (zOTUs) using the bioinformatic pipeline UNOISE3 (Edgar 2016a, 2016b). zOTUs were taxonomically classified using the Ribosomal Database Project Bayesian classifier in MOTHUR (Schloss 2020; Schloss et al. 2009).

#### 2.4.1 Alpha Diversity

Prior to analysis, some samples were discarded due to irregular sequencing coverage (Supporting Information Figure 1).

Data were filtered by prevalence as follows: OTUs were retained if 10% of their values contained at least 2 counts (Dhariwal et al. 2017). Data were rarefied to 1000 using the *phyloseq* package (Mcmurdie and Holmes 2013) to account for sequencing and sampling biases (Cameron et al. 2021). The alpha diversity of the gut microbiota was characterised using total species richness and Shannon‐Wiener indices. One‐way ANOVA, with human population density (high, medium, and low) as a factor, and Tukey's HSD multiple comparison test, were used to investigate potential differences in the gut microbiome alpha diversity of pademelons and wallabies across the urbanisation categories.

#### 2.4.2 Beta Diversity

Microbiome datasets collected by high‐throughput sequencing (HTS) of 16S rRNA gene are generally treated as compositional due to the limitations imposed by the instruments (Gloor et al. 2017). Thus, a compositional analysis approach (CoDa) is used in this study (Gloor et al. 2016). This required the transformation of data prior to statistical analysis, then the use of classical multivariate statistics (Aitchison 1982) described in more detail below.

Zeros (non‐detections) were replaced with the probability that it was a false zero (false non‐detection) using a Bayesian‐multiplicative replacement strategy, specifically the *cmultrepl* function in the *zCompositions* package (Palarea‐Albaladejo and Martín‐Fernández 2015). Centred log‐ratio (clr) transformation was performed on the data using the function *clr* in the *compositions* package (Van Den Boogaart and Tolosana‐Delgado 2008). Beta diversity was measured using Aitchison distance (Aitchison 1982). Differences in microbiome community structure across the three urbanisation categories were evaluated for each species with perMANOVA using the *adonis2* function from the *vegan* package (Oksanen et al. 2015). Comparison between urbanisation levels was undertaken with pairwise perMANOVA using the package *pairwise.adonis* (Martinez Arbizu 2020).

Principal Coordinate Analysis (PCoA) was used to visualise the community composition at the different urbanisation levels (Gloor et al. 2017) using the *betadisper* function from the *vegan* package (Oksanen et al. 2015).

#### 2.4.3 Composition

Composition was assessed based on the relative abundance of taxa. For each species, bacterial community composition was summarised at the phylum and genus levels across urbanisation intensities using relative abundance plots. To improve interpretability, only dominant taxa were visualised, with phyla representing > 1% relative abundance included in phylum‐level summaries and the nine most abundant genera shown at the genus level.

Differential abundance testing was conducted for each species using the ANOVA‐Like Differential Expression 2 (*ALDEx2*) package in R to identify taxa whose relative abundance differed significantly across urbanisation intensity groups. ALDEx2 accounts for the compositional nature of microbiome sequence data through centred log‐ratio transformation and Monte Carlo sampling, and results were reported as estimated effect sizes of change (Fernandes et al. 2014). Only the most abundant taxa were considered to avoid overinterpretation of results.

## 3 Result

### 3.1 Alpha Diversity

**FIGURE 2:**
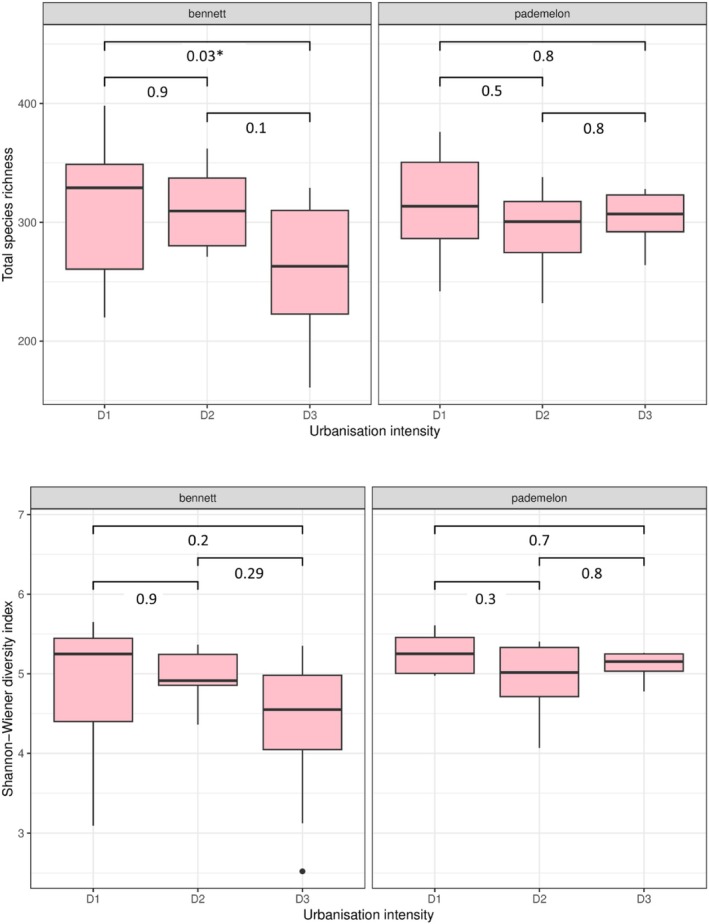
Box plots showing gut microbiome alpha diversity variation across the urbanisation gradient (D1 = high human density, D2 = medium human density and D3 = low human density) of the Bennett's wallaby (*N. r. rufogriseus*) and the Tasmanian pademelon ( *T. billardierii* ). Top: Total species richness. Bottom: Shannon‐Wiener diversity.

Our data generated 388,678 raw sequence reads across 57 macropod faecal samples. There was a significant difference in gut microbiome species richness between the urbanisation levels for Bennett's wallaby (*p* = 0.023), but not Tasmanian pademelon (*p* = 0.73) (Figure 2). The results of Tukey's HSD test showed that Bennett's wallabies from the most urbanised locations had lower total species richness compared to those from the least urbanised locations (Figure 2). The Shannon index was not significantly different for either of our species.

### 3.2 Beta Diversity

**FIGURE 3:**
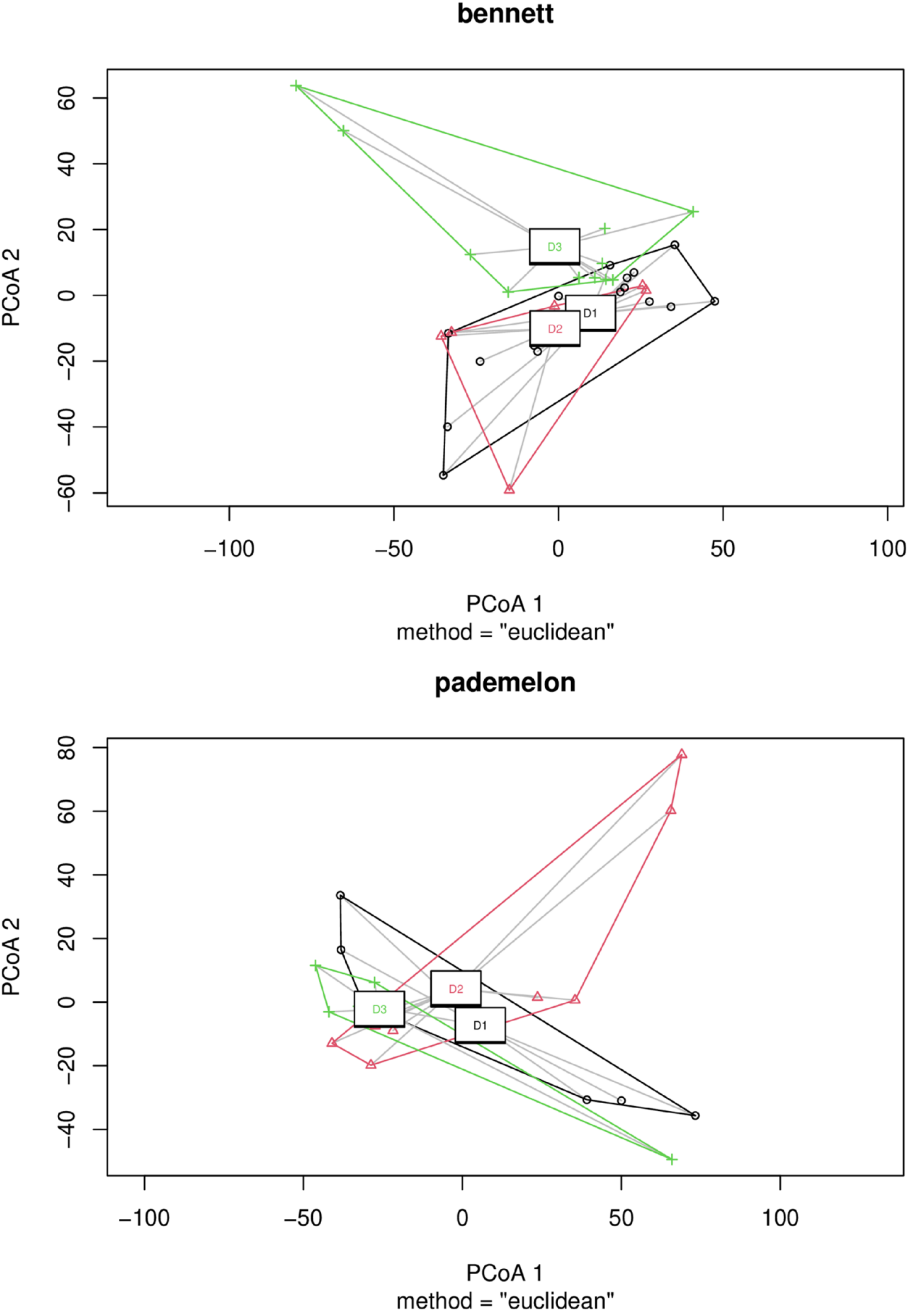
Multivariate dispersions as a measurement of gut bacterial communities for the Bennett's wallaby (*N. r. rufogriseus*) and the Tasmanian pademelon ( *T. billardierii* ) in Hobart, Tasmania, Australia. (D1 = high human density, D2 = medium density and D3 = low density).

Beta diversity, as measured by the Aitchison distance of samples, was significantly different across the levels of urbanisation for the Bennett's wallaby faecal samples (perMANOVA *p* = 0.006, Figure 3), but not for the Tasmanian pademelon samples, though this analysis was trending towards significance *p* = 0.09. Group dispersion of both species (Figure 3) was homogeneous (ANOVA *p* > 0.05), confirming that the significant dispersion is inter‐group. Pairwise perMANOVA results demonstrated a significant difference in gut beta diversity between Bennett's wallaby living at the highest and lowest urbanisation level (*p* = 0.012; Supporting Information Table 1, Appendix A).

### 3.3 Microbiome Composition

#### 3.3.1 Bennett's Wallaby

**FIGURE 4:**
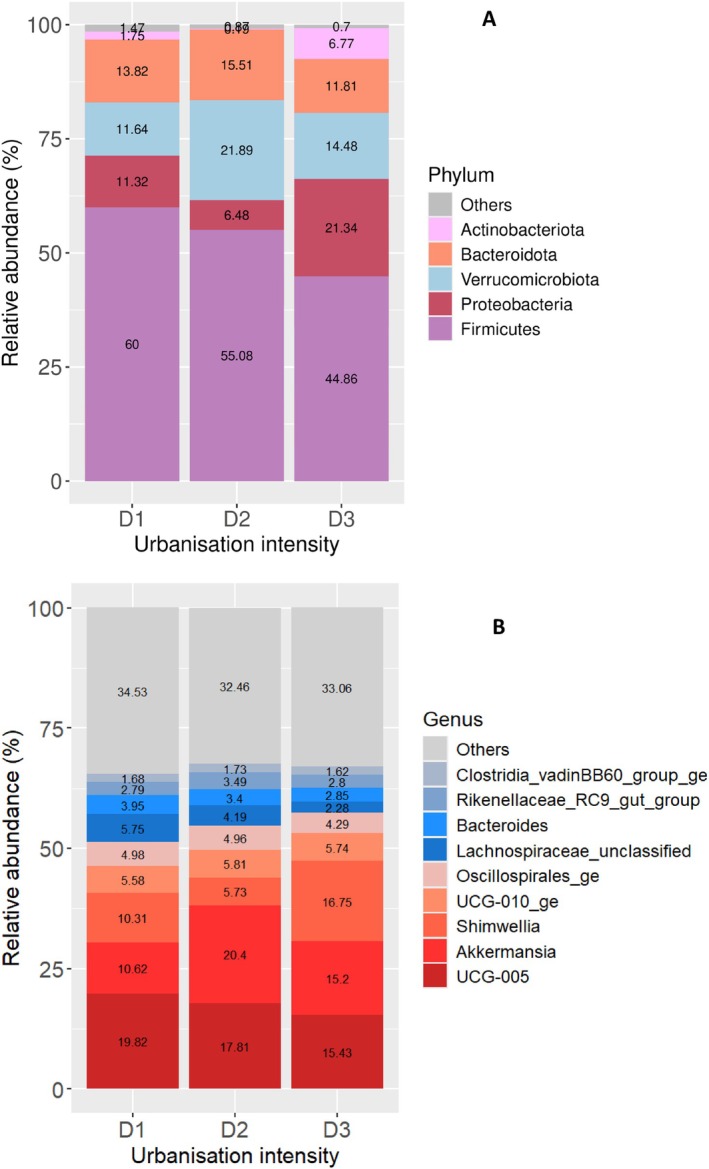
Gut microbial community composition of the Bennett's wallaby (*N. r. rufogriseus*) at (A) phylum level and (B) genus level across different levels of urbanisation (D1 = high human density, D2 = medium density and D3 = low density).

Firmicutes were the most relatively abundant phylum in the gut microbiome of the Bennett's wallabies (Figure 4A). Actinobacteriota was only prominently featured at the most rural sites, being the third most abundant phylum in animals living there (6.77%). Samples from the mid‐urbanisation level had the highest percentage of Verrucomicrobiota (21.89%) and the lowest percentage of Proteobacteria (6.48%).

We observed 3060 genera of bacteria across the 40 Bennett's wallaby faecal samples. The two most dominant bacteria at all the urbanisation levels were also *Ruminococcaceae UCG‐005* and *Akkermansia* sp., with *Akkermansia* sp. being the most abundant in animals living at the medium urbanisation areas (20.4%, Figure 4B).

The ALDEx2 results showed a slightly higher Verrucomicrobiota abundance in D2 compared to D1 (Supporting Information Figure 2C). *Lachnospiraceae_unclassified* abundance was lower in D3 compared to D1 (Supporting Information Table 2). There were no other major compositional differences across the levels of urbanisation for the wallaby (Supporting Information Figure 2 and Table 2).

#### 3.3.2 Tasmanian Pademelon

**FIGURE 5:**
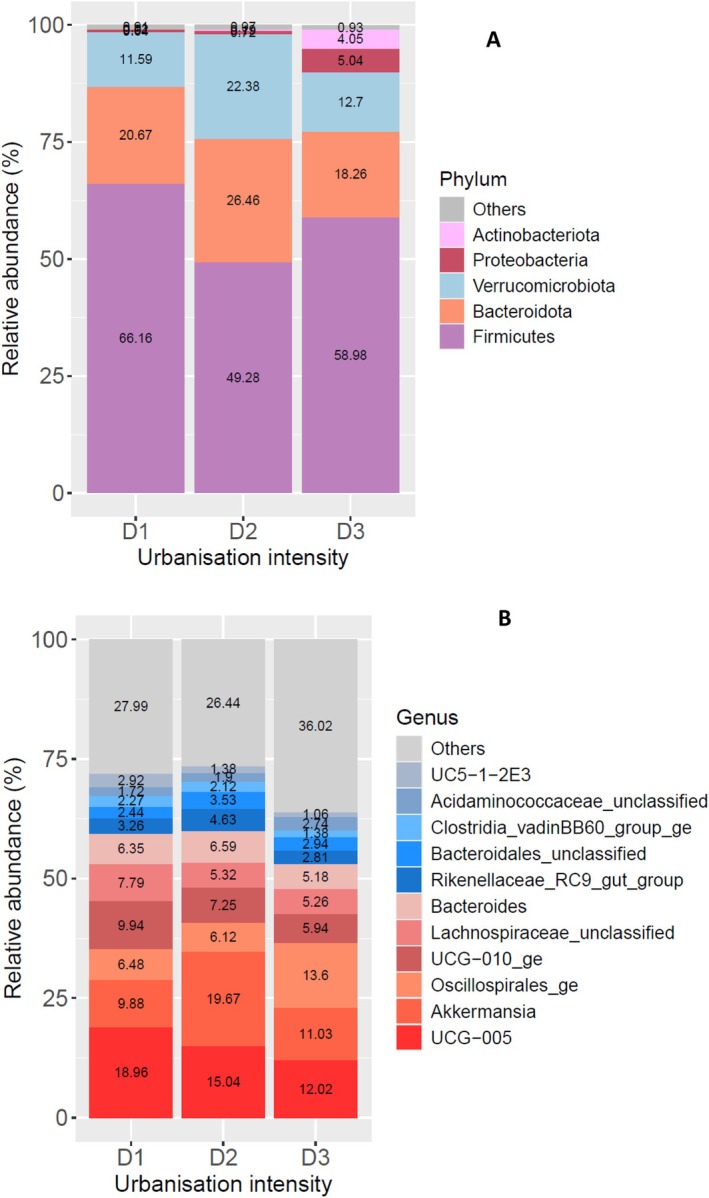
Gut microbial community composition of the Tasmanian pademelon ( *T. billardierii* ) at (A) phylum level and (B) genus level across different levels of urbanisation (D1 = high human density, D2 = medium density and D3 = low density).

The pademelon samples exhibited some similar compositional trends to the Bennett's wallaby samples. Firmicutes is also the phylum with the greatest overall relative abundance in the gut microbiome of the Tasmanian pademelon, and the most abundant at all intensities of urbanisation. It is the least abundant at the medium urbanisation level at ~50% compared to ~60% of the other two levels (Figure 5A). Bacteroidota were the second most abundant phylum overall, followed closely by Verrucomicrobiota. Actinobacteriota were more abundant in samples from animals living at the lowest level of urbanisation while Firmicutes showed a small increase in the most urban areas (Figure 5A, Supporting Information Figure 3).

Across the 24 pademelon faecal samples, 988 genera of bacteria were observed. At the genus level, the two most dominant bacteria in the pademelon's gut microbiome at all the urbanisation levels were *Ruminococcaceae UCG‐005* and *Akkermansia* sp. (Figure 5B).

The ALDEx2 result showed *Ruminococcaceae UCG‐005, Lachnospiraceae_UCG.010*, and *Oscillospiraceae_unclassified* abundances increased with urbanisation levels, whereas *Oscillospirales_ge* was more abundant in the animals living at the most rural areas (Figure 5B, Supporting Information Table 3).

## 4 Discussion

This study represents one of the first attempts to understand the pattern of macropod gut flora across an urbanisation level as defined by human population density, as well as one of the first to assess the differences in wildlife gut flora between categories of urbanisation in a small city. The results indicate that the alpha diversity was significantly lower in the most urbanised areas and the beta diversity was also significantly different between the most urbanised and the other areas. We also found that the community composition of the gut microbiome in both the Tasmanian pademelon and Bennett's wallaby differed along the urbanisation gradient.

Both alpha and beta diversity of the Bennett's wallaby faecal microbiome were found to be significantly different along the urban gradient defined in our study while the diversity of the pademelon gut microbiome displayed no significant differences. This difference between the species might be due to the different foraging habits adapted by these species in urban settings. For example, in a plantation habitat, Tasmanian pademelon were observed to prefer forbs when possible but would also eat grasses, while the opposite was true for Bennett's wallaby (Sprent and Mcarthur 2002).

On the other hand, while the perMANOVA indicated a non‐significant result for the pademelon's gut beta diversity, its marginal *p*‐value (Supporting Information Table 2, Appendix A) and the trend observed on the PCoA (Figure 3) implied that there was a trend and that with a bigger sample size, significant differences might have been detected. Note that our pademelon sample was smaller than the wallaby due to low‐quality sequence results. The connection between gut beta diversity and health conditions still needs to be explored further in the current literature for wildlife studies, as there were findings where it wasn't a predictive feature of health status (Fonseca et al. 2024), but also where it was associated with obesity (Lin et al. 2015). Thus, there is a range of mechanistic and field evidence to suggest that lower gut beta diversity, as we observed in the most highly urbanised wallaby gut microbiomes, could contribute to disease and poorer body condition in the urban populations of these macropods, but further research would be required.

This study's findings, with respect to wildlife gut microbiome composition, have some similarities with trends found in other studies. The gut microbiome of herbivores is highly specialised to break down cellulose and absorb nutrition from a fibrous, low nutrient diet (Zhu et al. 2011; Ilmberger et al. 2014), and is usually found to be dominated by Firmicutes and Bacteroidetes (Kohl and Dearing 2012; Fu et al. 2021; Sun et al. 2024). Similarly, our results show that Firmicutes is the most abundant phylum in the gut microbiome of both the Tasmanian pademelon and the Bennett's wallaby. The abundance of Firmicutes across both our study species is consistent with past studies on the gut flora of the wild eastern grey kangaroo ( *Macropus giganteus* , 47.39%) (O'Dea et al. 2021), and red kangaroo (*Osphranter rufus*, 54.26%) (Gulino et al. 2013).

In herbivores living in non‐urban habitats, an increase of Firmicutes abundance in gut flora has been associated with increased intake of grasses (more celluloid), and decrease when animals had more access to fruit (Baniel et al. 2021; Springer et al. 2017; Fu et al. 2021). In pademelon, this pattern is reflected in the decrease of *UCG‐005 in correspondence with the decrease of urbanisation intensity*. This is an important bacterium involved in the degradation of cellulose and hemicellulose (La Reau et al. 2016; Zhang et al. 2022). A related genus, *Oscillospira*, was found to decrease in abundance when fermented feed was added to the diets of geese (Yan, Zhou, et al. 2019). Pademelon living at the lowest level of urbanisation has a higher abundance of *Oscillospirales_ge*. The role of this genus in herbivores is not clear, but it has been found to increase in the absence of *Streptococcus* pathogen in chicken (Adewole and Akinyemi 2021). In pigs, these bacteria showed sensitivity to diet changes (Suárez‐Mesa et al. 2025). Both pademelon and wallaby showed decreases of Lachnospiraceae abundances in the highest level of urbanisation compared to the lowest. These bacteria are commonly linked to the degradation of dietary cellulose and polysaccharides in herbivores (Bai et al. 2018). They can be lost in the gut microbiome when the host is exposed to low‐fibre diets (Liu et al. 2026).

Additionally, in our study, Actinobacteria were more abundant in pademelon at the least urbanised sites, while Verrumicrobiota was moderately more abundant in wallaby at the medium urbanised areas. Actinobacteria usually assist hosts in digestion of complex carbohydrates, including plant‐derived oligosaccharides (Lewin et al. 2016), and contribute to maintenance of gut homeostasis (Binda et al. 2018). Typically, Verrucomicrobiota is a core phylym that's usually found in relatively low abundance in herbivore gut microbiomes (Newsome et al. 2020; Tong et al. 2022); here we found a small increase in abundance in wallabies living at the medium urban sites. Some genus belonging to this phylum seem to play a role in improving glucose metabolism and host's immune system (David et al. 2014; Costantini et al. 2017).

Previous studies have found that marsupials in urban environments often experience hardship like habitat fragmentation, leading to restriction in foraging movement and reduction in vegetation variety (Fardell and Dickman 2023). However, urban marsupials are also supplied with a stable source of human food, which they have been observed to access (Fardell and Dickman 2023). Fu et al. (2021) found Verrucomicrobiota and Actinobacteria to increase during the months associated with higher crude protein, crude fat and total sugar in the herbivorous diet of plateau pika ( *Ochotona curzoniae* ) and yak ( *Bos grunniens* ). Our findings might indicate that Bennett's wallaby and Tasmanian pademelon are exposed to a lower fibre diet when living in habitats with higher level of urbanisation. While less ideal than their natural non‐urban environment, more urbanised areas may provide pademelons and wallaby with access to higher protein, fat and sugar‐heavy human food. Thus, they might choose to supplement their own diet with high‐energy human food at higher levels of urbanisation. On the other hand, the medium urbanisation levels of a small city (50–1000 people/km^2^), may provide access to fruit and vegetables from gardens and compost bins than areas of higher urbanisation (Fardell and Dickman 2023).

## 5 Limitations

Our study adds another component of evidence to the sometimes‐conflicting research base on the impacts of urbanisation on wildlife microbiomes. The study was limited by the lack of negative controls to account for soil contamination. However, the sampling methods (protective gear usage, equipment sterilisation after each collection, taking the middle parts of the pellets for DNA extraction) and the overall abundance of microbes from a faecal sample should have limited the influence of the soil on the observed faecal microbiomes. The opportunistic nature of our collection method and low‐quality sequence results of some samples also lead to uneven sample numbers between sites. A bigger sample size could provide more information. There is also the possibility that some faecal samples were misclassified between these two species though size, morphology and consistency differences of their faeces and use of the PooFlip guidebook make this an unlikely impact. Finally, the faecal samples were collected across sites throughout the day, which meant that samples collected earlier in the day may have been fresher than samples collected later in the day. Collecting the sample from the middle of a faecal pellet may have accounted for some of this bias but it would not control for it entirely. Overall, there was a small number of samples in this study and there was a mismatch in the number of samples between wallaby (*n* = 40) compared to pademelon (*n* = 24) which may have influenced the power, especially for the pademelon analysis. All of these factors may have impacted our results.

## 6 Conclusion

Our study is one of the first studies of urban macropod gut flora, naming the red‐bellied pademelon ( *Thylogale billardierii* ) and the red‐necked wallaby (*Notamacropus rufogriseus rufogriseus*). There is some evidence of urbanisation's impacts on the macropod microbiome, although there appear to be nuances across species and some non‐linear relationships along a human population density gradient.

The need to understand the influence of cities and city living on the health of urban wildlife inhabitants is a topic of growing importance. Animal microbiomes, which are associated with and contribute to the overall health of the animal host, are a key knowledge gap linking urbanisation and biodiversity. As the urban environment is a complex mosaic made up of many different environments (Hahs and Mcdonnell 2006), and the microbiome is a complex system made up of many species each of which react differently to external pressure, it will take a considerable research effort with careful planning to resolve the various factors that may determine the interactions between urbanisation, microbiota and animal health. In particular, systematic investigation at different levels of urbanisation and different city sizes to explore multiple taxonomic host groups is required to unravel these complex interactions.

## Author Contributions

**Hanh K. D. Nguyen:** conceptualization, investigation, writing – original draft, writing – review and editing, formal analysis, funding acquisition, visualization. **Penelope J. Jones:** conceptualization, methodology, validation, writing – review and editing, supervision. **Dave Kendal:** writing – review and editing, investigation, conceptualization, methodology, supervision. **Shane M. Powell:** writing – review and editing, supervision, formal analysis. **Kelsie Raspin:** writing – review and editing, formal analysis, methodology. **Jo Dickinson:** writing – review and editing. **Emily J. Flies:** writing – review and editing, investigation, supervision, methodology, conceptualization.

## Funding

This project was funded by the Holsworth Wildlife Research Endowment—Equity Trustees Charitable Foundation & the Ecological Society of Australia.

## Ethics Statement

The research associated with this thesis abides by the international and Australian codes on research involving animals, the guidelines by the Australian Government's Office of the Gene Technology Regulator and the rulings of the Safety, Ethics and Institutional Biosafety Committees of the University.

## Conflicts of Interest

The authors declare no conflicts of interest.

## Supporting information


**Supporting Information Figure 1:** Rarefaction curves expressed as a function of DNA sequencing depth.
**Supporting Information Figure 2:** Differential abundance analysis at phylum level for Bennett's wallaby (*N. r. rufogriseus*). Largest ALDEx2 effect sizes of difference in centre log ratio transformed gut microbiome abundances between (A) D2 and D3, (B) D1 and D3, and (C) D1 and D2.
**Supporting Information Figure 3:** Differential abundance analysis at phylum level Tasmanian pademelon ( *T. billardierii* ). Largest ALDEx2 effect sizes of difference in centre log ratio transformed gut microbiome abundances between (A) D2 and D3, (B) D1 and D3, and (C) D1 and D2.
**Supporting Information Table 1:** Results of the pairwise perMANOVA showing the differences in faecal beta diversity of Bennett's wallaby and Tasmanian pademelon at different urbanisation level (D1 = high human density, D2 = medium density and D3 = low density).
**Supporting Information Table 2:** Differential abundance analysis at genus level of the Bennett's wallaby (*N. r. rufogriseus*). Only genera with the largest ALDEx2 effect sizes of difference in centre log ratio transformed were included (effect size > 0.9), along with the 95% CI for effect sizes as calculated by ALDEx2. Highlighted are the genera that are the most abundant (Figure 5).
**Supporting Information Table 3:** Differential abundance analysis at genus level of the Tasmanian pademelon ( *T. billardierii* ). Only genera with the largest ALDEx2 effect sizes of difference in centre log ratio transformed were included (effect size > 0.9), along with the 95% CI for effect sizes as calculated by ALDEx2. Highlighted are the genera that are the most abundant (Figure 5).

## Data Availability

The data that support the findings of this study are available on request from the corresponding author. The data are not publicly available due to privacy or ethical restrictions.
