# Supplementary material for: A Comparison of the Gut Microbiome of Two Sympatric Macropods Along an Urbanisation Gradient in Tasmania
Source: Environ Microbiol Rep. 2026 May 12;18(3):e70359. doi: 10.1111/1758-2229.70359 (PMC13162132; doi:10.1111/1758-2229.70359)
Supplement: Supplementary file 1 — Supporting Information Figure 1: Rarefaction curves expressed as a function of DNA sequencing depth. Supporting Information Figure 2: Differential abundance analysis at phylum level for Bennett's wallaby (N. r. rufogriseus). Largest ALDEx2 effect sizes of difference in centre log ratio transformed gut microbiome abundances between (A) D2 and D3, (B) D1 and D3, and (C) D1 and D2. Supporting Information Figure 3: Differential abundance analysis at phylum level Tasmanian pademelon ( T. billardierii ). Largest ALDEx2 effect sizes of difference in centre log ratio transformed gut microbiome abundances between (A) D2 and D3, (B) D1 and D3, and (C) D1 and D2. Supporting Information Table 1: Results of the pairwise perMANOVA showing the differences in faecal beta diversity of Bennett's wallaby and Tasmanian pademelon at different urbanisation level (D1 = high human density, D2 = medium density and D3 = low density). Supporting Information Table 2: Differential abundance analysis at genus level of the Bennett's wallaby (N. r. rufogriseus). Only genera with the largest ALDEx2 effect sizes of difference in centre log ratio transformed were included (effect size > 0.9), along with the 95% CI for effect sizes as calculated by ALDEx2. Highlighted are the genera that are the most abundant (Figure 5). Supporting Information Table 3: Differential abundance analysis at genus level of the Tasmanian pademelon ( T. billardierii ). Only genera with the largest ALDEx2 effect sizes of difference in centre log ratio transformed were included (effect size > 0.9), along with the 95% CI for effect sizes as calculated by ALDEx2. Highlighted are the genera that are the most abundant (Figure 5). [file EMI4-18-e70359-s001.docx]

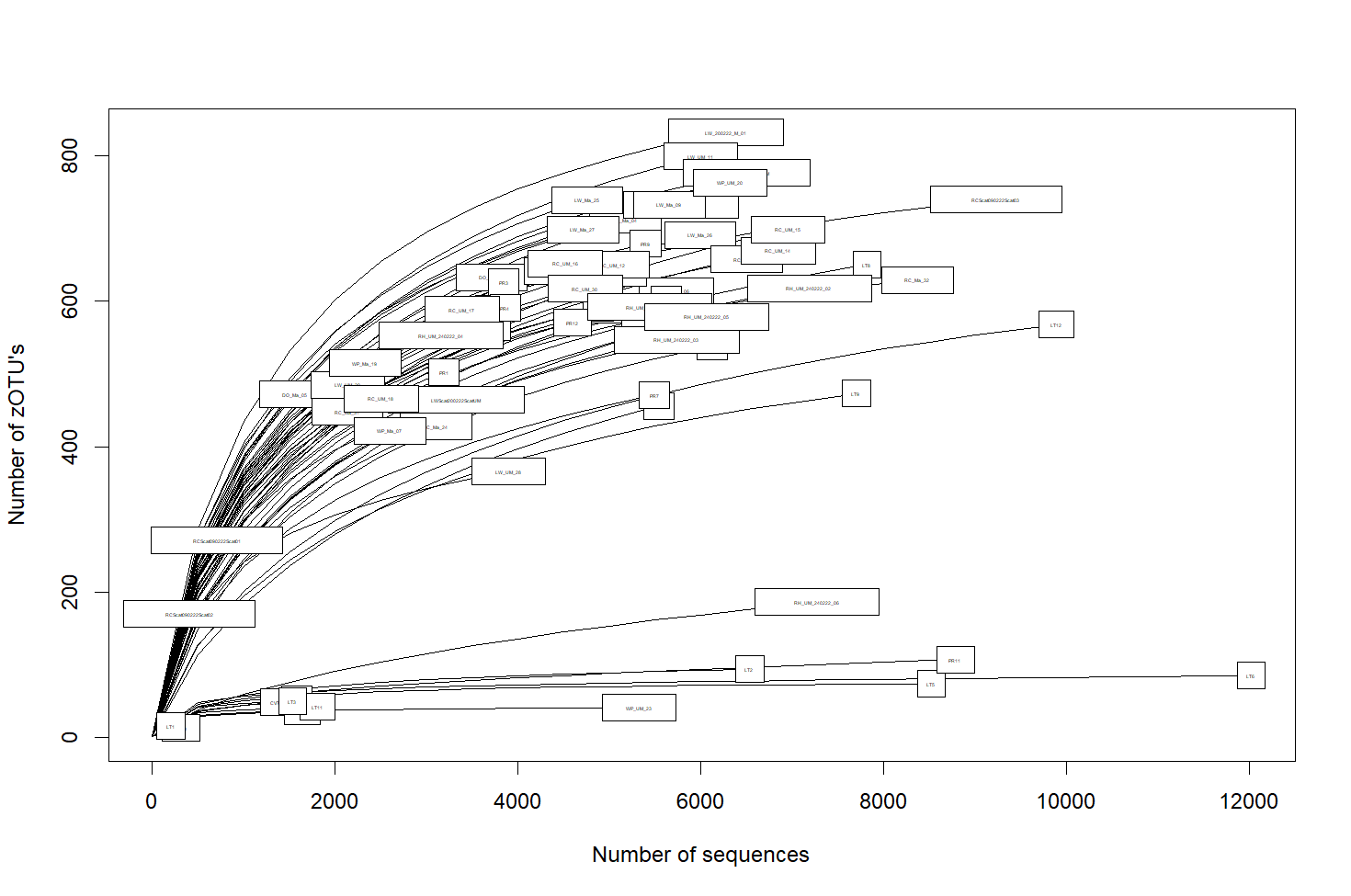


**Supplementary Figure 1.** Rarefaction curves expressed as a function of DNA sequencing depth


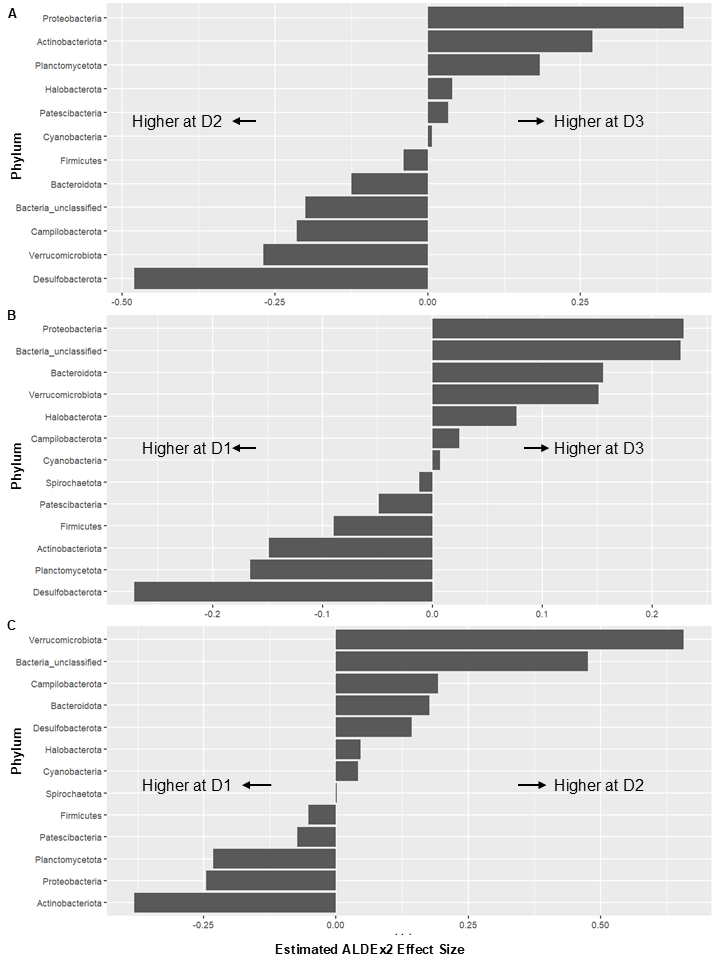


**Supplementary Figure 2.** Differential abundance analysis at phylum level for Bennett’s wallaby (N. r. rufogriseus). Largest ALDEx2 effect sizes of difference in centre log ratio transformed gut microbiome abundances between A) D2 and D3, B) D1 and D3, and C) D1 and D2.

(D1 = high human density, D2 = medium density and D3 = low density).


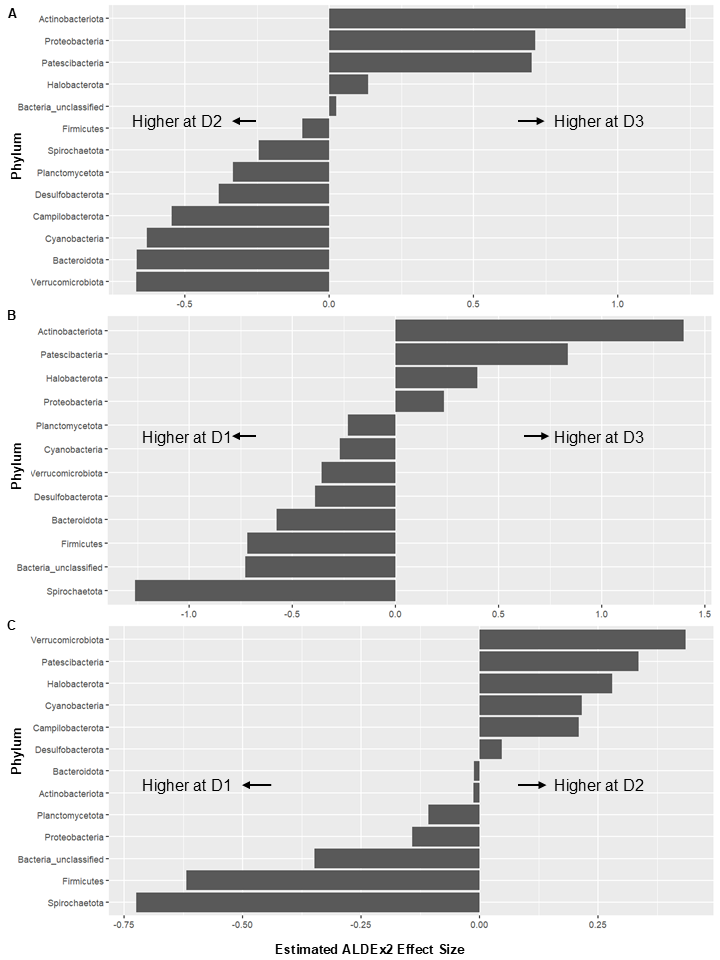


**Supplementary Figure 3** Differential abundance analysis at phylum level Tasmanian pademelon (T. billardierii). Largest ALDEx2 effect sizes of difference in centre log ratio transformed gut microbiome abundances between A) D2 and D3, B) D1 and D3, and C) D1 and D2.

(D1 = high human density, D2 = medium density and D3 = low density).

**Supplementary Table 1.** Results of the pairwise perMANOVA showing the differences in faecal beta diversity of Bennett’s wallaby and Tasmanian pademelon at different urbanisation level (D1 = high human density, D2 = medium density and D3 = low density)

| Species | perMANOVA | Pairwise perMANOVA | | |
| --- | --- | --- | --- | --- |
|  |  | D1 vs D2 | D2 vs D3 | D1 vs D3 |
| Bennett’s wallaby | 0.006* | 0.5 | 0.09. | 0.02* |
| Tasmanian pademelon | 0.095 | 0.9 | 0.38 | 0.35 |

**Supplementary Table 2**. Differential abundance analysis at genus level of the Bennett’s wallaby (*N. r. rufogriseus*). Only genera with the largest ALDEx2 effect sizes of difference in centre log ratio transformed were included (effect size > 0.9), along with the 95%CI for effect sizes as calculated by ALDEx2. Highlighted are the genera that are the most abundant (Fig.5).

D1 = high human density, D2 = medium density and D3 = low density

| **Genus** | **Effect size** | **effect.low** | **effect.high** | **Note** |
| --- | --- | --- | --- | --- |
| Comparison: D1 to D3 | | | | |
| Lachnospiraceae_unclassified | -1.42821 | -8.86793 | 0.486109 | Higher in D1 |
| Comparison: D2 to D3 | | | | |
| WCHB1.41_ge | -1.34663 | -9.33353 | 0.25431 | Higher in D2 |

**Supplementary Table 3.** Differential abundance analysis at genus level of the Tasmanian pademelon (*T. billardierii*). Only genera with the largest ALDEx2 effect sizes of difference in centre log ratio transformed were included (effect size > 0.9), along with the 95%CI for effect sizes as calculated by ALDEx2. Highlighted are the genera that are the most abundant (Fig.5).

D1 = high human density, D2 = medium density and D3 = low density

| **Genus** | **Effect size** | **effect.low** | **effect.high** | **Note** |
| --- | --- | --- | --- | --- |
| Comparison: D1 to D3 | | | | |
| Flavonifractor | -1.62817 | -9.33034 | -0.16764 | Higher in D1 |
| Alistipes | -1.2831 | -8.68636 | 0.308529 | Higher in D1 |
| UCG.005 | -1.07623 | -8.20347 | 0.785871 | Higher in D1 |
| Lachnospiraceae_UCG.010 | -1.05569 | -7.88769 | 0.885588 | Higher in D1 |
| Oscillospiraceae_unclassified | -0.9331 | -11.5804 | 0.722011 | Higher in D1 |
| Muribaculaceae_unclassified | -0.93275 | -7.77502 | 1.573319 | Higher in D1 |
| Oscillospirales_ge | 0.904359 | -1.70318 | 9.50646 | Higher in D3 |
| Lysinibacillus | 0.907586 | -1.6317 | 7.601014 | Higher in D3 |
| Bacillaceae_unclassified | 0.963037 | -1.16456 | 8.975274 | Higher in D3 |
| Neomicrococcus | 0.96428 | -1.08627 | 8.886628 | Higher in D3 |
| Candidatus_Saccharimonas | 1.020511 | -0.67777 | 10.07401 | Higher in D3 |
| Caryophanon | 1.157167 | -1.6484 | 10.08526 | Higher in D3 |
| Acinetobacter | 1.2229 | -1.14224 | 8.994396 | Higher in D3 |
| Micrococcaceae_unclassified | 1.354301 | -1.11477 | 14.58358 | Higher in D3 |
| Psychrobacillus | 1.388478 | -1.24795 | 16.78266 | Higher in D3 |
| Planococcaceae_unclassified | 1.55072 | -1.67173 | 19.47353 | Higher in D3 |
| Comparison: D1 to D2 | | | | |
| Alistipes | -1.26895 | -7.23056 | 0.616265 | Higher in D1 |
| Bacteroidales_RF16_group_ge | 0.949207 | -0.55134 | 13.13959 | Higher in D2 |
| Comparison: D2 to D3 | | | | |
| Bacteroidales_RF16_group_ge | -2.05873 | -16.8948 | -0.06356 | Higher in D2 |
| Rikenellaceae_RC9_gut_group | -0.98962 | -10.1528 | 1.34638 | Higher in D2 |
| Flavonifractor | -0.97279 | -6.97896 | 0.874956 | Higher in D2 |
| Lachnospiraceae_NK3A20_group | -0.9574 | -10.6975 | 1.023632 | Higher in D2 |
| Oscillospirales_ge | 0.904791 | -0.64058 | 8.875652 | Higher in D3 |
| Monoglobus | 0.923389 | -0.94745 | 8.678657 | Higher in D3 |
| Clostridia_UCG.014_ge | 0.976953 | -0.99971 | 7.228626 | Higher in D3 |
| Bacillaceae_unclassified | 0.978335 | -1.45235 | 7.893023 | Higher in D3 |
| Acinetobacter | 1.096872 | -0.90065 | 9.272812 | Higher in D3 |
| Caryophanon | 1.114583 | -1.29457 | 7.062143 | Higher in D3 |
| Micrococcaceae_unclassified | 1.154044 | -1.74449 | 12.72609 | Higher in D3 |
| Psychrobacillus | 1.160721 | -1.51232 | 12.19027 | Higher in D3 |
| Planococcaceae_unclassified | 1.415096 | -1.09582 | 19.55456 | Higher in D3 |
